# Commentary: Impact of extended reality (XR) simulation on ophthalmology training outcomes: an updated systematic review and meta-analysis

**DOI:** 10.3389/fmed.2026.1906140

**Published:** 2026-09-16

**Authors:** Van Charles Lansingh, Stephen Chai, Nicoletta Fynn-Thompson, Akshay G. Nair, Jared Tuttle

**Affiliations:** 1 HelpMeSee, Jersey City, NJ, United States; 2 Instituto Mexicano de Oftalmología, Santiago de Querétaro, Qro, Mexico; 3 Department of Public Health Sciences, University of Miami Miller School of Medicine, Miami, FL, United States; 4 Department of Ophthalmology and Visual Sciences, John A. Moran Eye Center, University of Utah, Salt Lake City, UT, United States; 5 Centro de Investigación en Salud Poblacional, Instituto Nacional de Salud Pública, Cuernavaca, Morelos, Mexico; 6 California University of Science and Medicine, Colton, CA, United States; 7 Ophthalmic Plastic Surgery and Ocular Oncology Services, Dr Agarwal’s Group of Eye Hospitals, Mumbai, India; 8 HMS Vision Pvt. Ltd., Mumbai, India; 9 University of Texas Health Science Center at San Antonio, San Antonio, TX, United States

**Keywords:** ophthalmology education, simulator validation, surgical simulation, virtual reality simulation, virtual reality simulators

## Introduction

Simulation-based training is an important component of ophthalmology training because it offers trainees repeatable practice while maintaining patient safety. The Accreditation Council for Graduate Medical Education (ACGME) requires ophthalmology residency programs to provide surgical skills development resources in the form of wet labs, dry labs, or simulation labs. Since the introduction of virtual reality (VR) simulators in the mid-2000s, residency programs have increasingly incorporated them into ophthalmology training curricula, with a growing number of studies evaluating curricula standardization and trainee outcomes (1). We read with great interest the article by Jiang Y et al. (2) evaluating the literature surrounding virtual reality simulation and ophthalmology training outcomes and commend the authors for presenting a timely systematic review and meta-analysis on the topic. However, as VR simulators continue to strive towards standardization, greater consistency in the definitions and methodologies used to evaluate these technologies is necessary.

## Terminological precision

With the growing application of different modalities of immersive technologies in ophthalmology education, proper use of standardized terminology is crucial. Jiang Y et al. are concerned with extended reality simulation's impact on ophthalmology training outcomes and correctly define extended reality as encompassing VR and augmented reality (AR). However, the literature included in the review was predominantly focused on VR simulation, despite the study being framed around XR simulation. In the introduction, XR is broadly framed as a training modality that provides repeatable and measurable practice without patient risk. This is a description that has often only applied to VR simulation platforms specifically, rather than XR. Additionally, when the authors discuss how randomized controlled trials and prospective studies suggest that XR simulation improves technical performance, with higher scores and fewer errors on simulators and, in some settings, during live surgery, the cited studies utilized VR simulation rather than mixed reality (MR) or AR in their study design (3, 4). Topics such as simulator survey data, barriers to acquisition, and standardization are also supported by studies focused primarily on VR simulators. Thus, when referencing individual studies that specifically evaluate a single modality, the term XR should not be used to broadly describe their results. It is important to note that we are not necessarily arguing against the use of the term XR itself, but rather for its use in the appropriate context. XR has been utilized for purposes beyond procedural simulation, including visual impairment simulation to strengthen clinician education, vision rehabilitation, and understanding of functional vision (5). Given that this paper is primarily concerned with ophthalmology training outcomes, we believe that virtual reality is the more appropriate term in this context, as the systematic review predominantly evaluated studies involving VR simulators, with no mixed reality studies referenced and only a single augmented reality simulator included. We would advise future studies to use the term XR cautiously in the context of ophthalmology training outcomes until evidence that is broadly representative of VR, AR, and MR is available. Since methodological differences between studies have consistently limited broader comparative analysis in previous systematic reviews, terminological inconsistencies may further complicate such efforts (6).

## Implications of VR simulation study design

The authors correctly note that an important limitation of conducting a systematic review and meta-analysis on VR simulator training outcomes is the heterogeneity in assessment methods, outcomes measures, and curriculum design. However, further discussion regarding how studies quantify and define VR simulation training experience is warranted. This is because VR simulation training seeks to establish surgical competency based on proficiency rather than time alone, often incorporating factors such as expert supervision, structured feedback, and demonstrated performance rather than an hourly log. Therefore, when a study design is centered around time, it assumes that competence is tied to exposure, when research has shown that trainees acquire surgical skills at different rates (7). Additionally, studies have shown that ophthalmology training is positively impacted by components such as supervision. An example of a VR simulator that weaves such components into the simulator experience is the HelpMeSee simulator (HelpMeSee, Inc., Jersey City, NJ, USA) which utilizes high-fidelity VR with haptic feedback to deliver a standardized, instructor-led curriculum on cataract surgery. A randomized controlled trial among 19 novice residents showed that the simulation group had significantly fewer total and major errors in scleral tunnel construction during MSICS compared to controls (8). As a result, close attention must be paid to how studies measure simulation exposure among trainees. We applaud the authors for detailing the study interventions in Table 1, allowing readers to better understand whether trainees were exposed to simulation through hourly exposure alone or through structured curricula involving supervision, feedback, or specific training modules. However, simulation-based studies must be designed such that trainee interaction with the simulator reflects the manner in which the platform was intended to be used educationally, rather than reducing exposure to time-based metrics.

Indeed, the validity evidence supporting VR simulators and their associated assessment metrics must be considered and integrated into VR simulation studies. Face validity refers to whether a simulator appears realistic to users, content validity evaluates whether the simulator adequately represents the surgical tasks being taught, and construct validity assesses whether the simulator can distinguish between differing levels of surgical expertise. These distinctions are important because improvements in simulator-derived performance scores may not necessarily translate into clinically meaningful surgical competency without robust validity evidence (9).

## Discussion

Despite these limitations, Jiang et al. provide a valuable systematic review and analysis of the literature on cataract surgery simulator training and surgical outcomes. Simulation has become an inseparable component of cataract surgical training, and studies evaluating VR simulator curricula and surgical outcomes are continuing to grow in volume and quality. Thus, we should aim to be as specific as possible when using simulation terminology and when discussing study methodology. In doing so, future comparative analyses may allow for more meaningful conclusions and help programs determine which components of simulation technology should be incorporated in cataract surgical training curricula.
